# Late Onset Hearing Loss in Very Low Birth Weight Infants

**DOI:** 10.21203/rs.3.rs-4249951/v1

**Published:** 2024-04-22

**Authors:** Lynn Iwamoto, Chloe Anne Liu

**Affiliations:** University of Hawaii John A Burns School of Medicine; University of New Mexico

**Keywords:** Late onset hearing loss, delayed onset hearing loss, conductive hearing loss, NICU, Very Low Birth Weight Infants

## Abstract

**Objective:**

To determine the incidence of late onset hearing loss and associated risk factors in very low birth weight (VLBW) infants.

**Study Design:**

Retrospective study (2003–2015) of post-discharge hearing outcomes and risk factors in the VLBW infant population, before and after the institution of a standardized follow-up program.

**Results:**

Late onset hearing loss increased from 2.9 per 100 VLBW infants to 7.8 per 100 after instituting a monitoring protocol. The follow-up compliance rate nearly doubled. Both infants with late-onset sensorineural hearing loss and those with a conductive component were identified. The rate of conductive loss detection increased seven-fold.

**Conclusion:**

The institution of a standardized hearing follow-up program significantly increased the detection of late onset hearing loss in VLBW infants. A significant proportion of those with late onset hearing loss had a conductive component. Without identification and treatment, even conductive losses may negatively impact speech and language development.

## Introduction

Infants requiring admission to the Neonatal intensive care unit (NICU) are at a ten-fold higher risk for early onset sensorineural hearing loss (SNHL), as compared to well-baby nursery infants. This is a function of both inherent pre-disposing factors, as well as exposure to potentially ototoxic medications and treatments. Moreover, within the NICU population, very low birthweight (VLBW) infants are at a higher risk for hearing loss (HL), due to a high acuity of illness in association with a complicated and protracted hospital stay. We previously reported a five-fold increased prevalence of early-onset hearing loss in our VLBW infants, as compared to our non-VLBW NICU population (ref ^1^).

Although by protocol, NICU infants are routinely screened for hearing during their hospitalization, unfortunately, not all hearing impairment can be detected by hospital discharge. Hearing impairment originating during a NICU stay may take time to develop (ref ^2,3^). Thus, any infant passing the pre-discharge newborn hearing screen may present sometime later with HL. The outcome is that providers may miss infants with late or delayed onset loss without standardized post-discharge follow-up. National recommendations published by the Joint Committee on Infant Hearing (JCIH) in the year 2000 were accordingly developed to mitigate this potential adverse outcome (ref 4).

At the time, all infants admitted to the NICU for greater than two days were identified to be at risk for late onset loss with recommendations to monitor post-discharge at 6-month intervals. This amounted to a very large number of formal evaluations, since it was applicable to nearly all NICU infants. However, formal audiological testing that is nearly universally applied may be challenging, expensive, and may overwhelm pediatric audiology resources. To address these concerns, JCIH guidelines were modified in 2007 decreasing recommendations to a minimum of one post-discharge evaluation by 24 to 30 months of age (ref 5). This recommendation did not factor in the level of ototoxic risk, with potential delayed detection and intervention well beyond the recommended 6 months of age. Therefore, in 2010 we established a standardized protocol to gauge the timing and frequency of monitoring based on identifiable risk factors.

In this study, the prevalence rates and types of late onset HL in the VLBW infant population are described before and after the 2007 changes to the JCIH guidelines for follow up. This is one of the few studies to investigate late onset HL outcomes and risk factors specific to the VLBW infant population. Our NICU serves as the neonatal component of the only regional perinatal center for the state of Hawaii, which includes the majority of the NICU follow-up and allows for closer follow-up compliance.

## Methods

This is a retrospective case-controlled study of VLBW infants, born at less than or equal to 1500 grams between the years of 2003–2015, who were both admitted and discharged from the NICU at Kapiolani Medical Center for Women and Children (KMCWC), the regional perinatal center, in Honolulu, Hawaii. This study was determined to be exempt from institutional review by the Hawaii Pacific Health Research Institute.

### Population: Late Onset HL Infants and Matched Controls:

**Figure 1:**
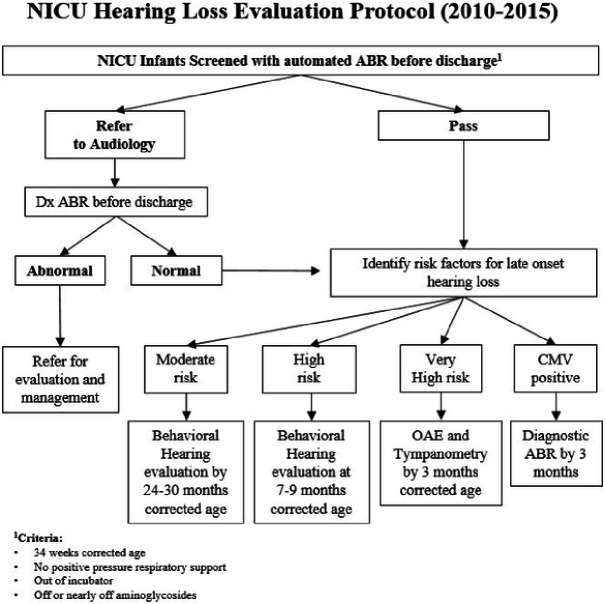
KMCWC NICU Protocol for late onset hearing loss risk assessment and follow up. Decision points and follow up recommendations for NICU infants screened for hearing loss before hospital discharge.

Late onset HL cases (n = 80) represents a population of infants who passed the screening auditory brainstem response (ABR) or did not pass but had normal hearing on the pre-discharge diagnostic ABR, who were subsequently diagnosed with hearing deficits by one-year corrected age. Control cases (n = 179) were VLBW NICU infants who passed the initial screening ABR as well as the follow-up exam resulting in the diagnosis of normal hearing by one-year corrected age. Controls were matched for birth weight, gestational age at birth, and year of birth. Two controls were identified for every infant with late onset HL. Infants with early onset loss, who failed the initial hearing screen with hearing loss confirmed by a diagnostic ABR prior to discharge, were excluded from this report (Fig. 1).

### Data Collection

Data extraction was done by manual chart review and compiled into spreadsheets. Demographic data collection included birth weight, gestational age, year of birth, and hospital length of stay. The hearing data consisted of newborn hearing screening results, discharge referrals, and behavioral hearing evaluation results including laterality, type, and degree of HL. Co-morbidities were also collected and analyzed, including intraventricular hemorrhage, chronic lung disease, retinopathy of prematurity, and necrotizing enterocolitis. Risk factors for late onset loss were those outlined in the 2007 JCIH recommendations, including significant ventilator support, severe hyperbilirubinemia, neurologic injury, in utero infections (cytomegalovirus [CMV] and toxoplasmosis), and exposure to ototoxic medications.

### NICU Hearing Screening:

Prior to discharge, hearing screening was performed for all infants using an automated ABR screener (ALGO 2E, Natus Medical, San Carlos, CA, USA). Testing is performed when NICU infants are at least 34 weeks corrected gestational age, off positive pressure respiratory support, out of an incubator, and no longer receiving aminoglycosides. Infants who did not pass were subsequently evaluated by diagnostic ABR (dxABR) prior to discharge to validate the screening results.

### Diagnostic Evaluation:

When an infant fails the automated newborn hearing screen, a diagnostic evaluation is performed prior to discharge to validate the screening result. This is standard practice in our island state with approximately 30% of the population living remotely on an outer island, lacking pediatric audiology services. The pre-discharge diagnostic evaluation includes the dxABR, distortion product otoacoustic emissions (OAE), and tympanometry. The dxABR is performed using the Bio-logic Navigator Pro AEP system (Natus Hearing Diagnostics, Bio-Logic Systems Corp, Mundelein, IL, USA) with the following parameters: 100 microseconds click stimulus, 33.3 clicks/sec repetition and tone burst at 27.1/sec repetition with Blackman ramp filtering. Responses above 20 dBnHL are considered abnormal. Recordings are made ipsilaterally using a single channel electrode montage. Click stimuli and tone bursts (500 Hz, 1000 Hz and 4000 Hz) are used. Distortion product OAE is measured using the Bio-logic Scout OAE system (Natus Hearing Diagnostics, Bio-Logic Systems Corp, Mundelein, IL, USA). A 750–8000 Hz diagnostic testing protocol is utilized. Tympanometry employs a 1 kHz probe tone (GSI Tympstar, Grason Stadler, Eden Prairie, MN, USA).

### Late Onset Hearing Loss Risk Factor Stratification:

As mentioned, we modified the JCIH 2007 recommendations to accomodate our population, resources, and geographic constraints. Four levels of risk factors were created. Post-discharge follow-up recommendations were stratified according to level of risk (Table 1). Highest risk infants with congenital CMV infection; were evaluated by dxABR by 3 months adjusted age. “Very high risk” infants with craniofacial anomalies (cleft palate, ear microtia or atresia) or bacterial meningitis; had physiologic evaluations by 3 months adjusted age. “High risk” infants were comprised of a heterogeneous variety of subgroups, those with a family history of childhood HL, those born at ≤ 30 weeks GA or with BW ≤ 1500 grams, or with conditions or interventions associated with HL (ECMO, other in-utero infections, syndromes associated with progressive HL, neurodegenerative disorders). These infants had behavioral hearing evaluations by 7 months adjusted age. The final group of infants at risk for HL were those who were hospitalized in the NICU for more than five days but lacked any other discrete risk factors. These infants were considered at “moderate” risk; with a behavioral hearing evaluation by 24–30 months and close surveillance for speech and language developmental milestones as per the 2007 JCIH guidelines.

### Behavioral Hearing Evaluation for Late Onset Hearing Loss:

Infants with identifiable risk factors for late onset HL were instructed to return for evaluation at 7–9 months corrected age for a behavioral hearing evaluation. Testing included hearing sensitivity evaluation by visual reinforcement audiometry, detection of cochlear transient evoked OAE, and middle ear evaluation by tympanometry. Visual reinforcement audiometry was completed in a sound booth using test stimuli at 0.5–4 kHz and speech awareness thresholds. Transient evoked OAE were recorded using the Otodynamics Echoport (Hatfield, Herts, UK). Tympanograms were obtained using a 226 Hz probe tones.

### Definitions of Sensorineural Hearing Loss (SNHL) and Conductive Hearing Loss (CHL):

SNHL was defined as HL in the mild range (30 dBnHL) or greater, along with normal tympanograms and detectable OAE. CHL was defined as mild range HL (30 dBnHL) or greater, with a conductive component (flat Type B tympanogram and absent OAE). However, for infants with CHL, an underlying SNHL component could not be confirmed since it is unlikely to distinguish mixed losses after a single post-discharge evaluation.

### Statistical Analysis:

Rates of protocol follow-up compliance were compared between cohorts of infants before and after 2010 when the standardized monitoring protocol was initiated and embedded into the electronic health record. Incidence rates of late onset HL were also compared pre- and post-protocol initiation. Infants identified with late onset HL were compared with matched controls for the association of specific risk factors. T-tests were performed for parametric data comparisons while either Chi-square analysis or Fisher exact testing was utilized to evaluate categorical data, depending on the size of the groups.

## Results

### Referral Documentation and Follow-up Compliance:

**Figure 2:**
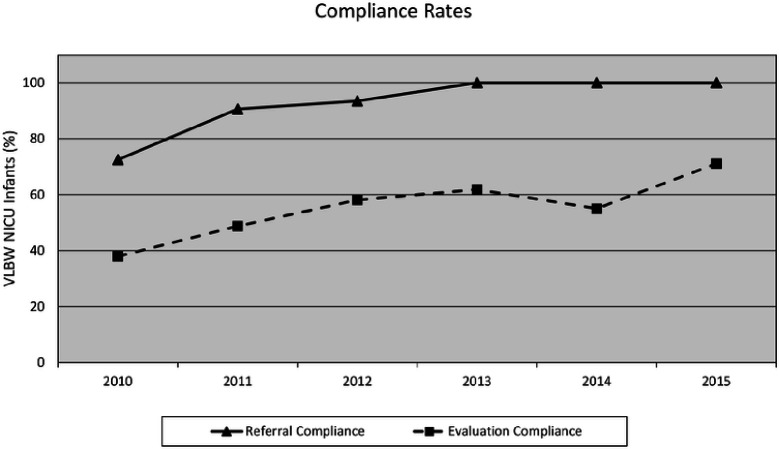
Compliance Rates for referral at discharge and completion of outpatient behavioral hearing evaluation after the initiation of a late onset hearing loss monitoring protocol in 2010.

During the study period, there were 1552 VLBW infants admitted to the NICU from 2003–2015 and screened for hearing prior to discharge: 837 infants were admitted between 2003–2009 (pre-standardization), and 715 infants between 2010–2015 (post-standardization). Overall, standardization resulted in increased referral compliance. For example, documentation of the referral in the discharge summary improved from a pre-standardization rate of 73% (n = 87 of 120) of infants discharged in 2010 to a post-standardization rate of 100% (n = 126) by 2013. This rate was maintained through 2015. As a result, the compliance rate for completed follow-up evaluations also increased from the disappointing annual rate of 38% (n = 33 of 120) in 2010 to 71% (n = 64 of 90) in 2015 (p < 0.0001) (Fig. 2).

### Late Onset Hearing Loss Incidence:

**Figure 3:**
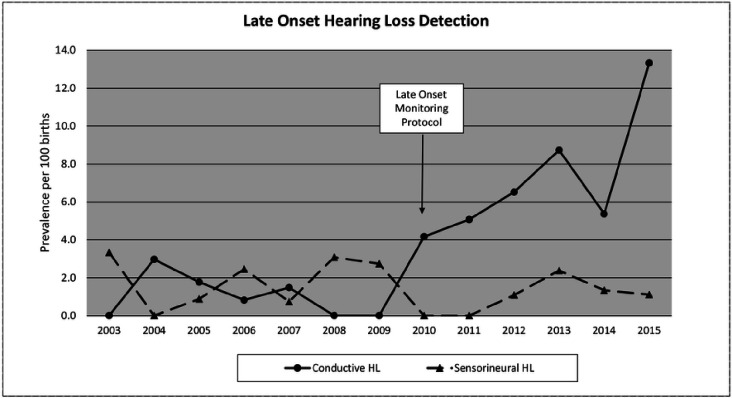
Incidence rates of conductive and sensorineural hearing loss detected in VLBW infants discharged from the KMCWC NICU before and after initiating a protocol to monitor for late onset hearing loss.

The increase in follow-up compliance translated to a significant increase in identification of infants with late onset HL. Hence, the protocol pre-standardization rate of 2.9/100 (n = 24 of 837) VLBW infants (2003–2009) increased to 7.8/100 (n = 56 of 715) post-standardization (2010–2015) (p = 0.003) (Fig. 3). Although the total rate of identified late onset hearing loss increased, the increase was inconsistent across types of HL. To be sure, there was a nearly seven-fold increase in the detection rate of CHL from pre-standardization (n = 8 of 837) to post-standardization (n = 49 of 715) (0.96 vs 6.85 per 100 VLBW infants respectively). Conversely, there was no significant change in the rate of SNHL from pre-(n = 16 of 837) to post-standardization (n = 7 of 715) (1.91 pre vs 0.98 post per 100 VLBW infants). Taken together, we documented an overall late onset prevalence rate of 1.5% for SNHL and 3.7% for CHL.

### Risk Factors:

Risk factors for late onset HL were compared between cases and matched controls. The cases and controls were matched for birth weight (BW) (1.095 ± 0.312 kg vs 1.112 ± 0.292 kg, p = 0.668) and gestational age (GA) (28.0 ± 2.9 wks vs 28.2 ± 2.8 wks, p = 0.535). In this study, congenital CMV infection (p = 0.001) was the only factor found to be associated with a significant risk of developing late onset HL (Table 2).

## Discussion

In this 13-year retrospective study, the implementation of a standardized protocol to monitor VLBW infants for late onset HL improved the percentage of at-risk infants screened and consequently increased the identification of infants with SNHL and CHL. This in turn should lead to appropriate and timely interventions to minimize language deficits and optimize developmental outcomes.

Preterm and VLBW neonates requiring admission to the NICU are at high risk for complications due to an immature system subjected to invasive medical, pharmacological, and technological therapies required to sustain the life of an infant transitioning to the harsh environment of the extrauterine world. Notably, VLBW infants may experience conditions that predispose them to hypoxia, hyperbilirubinemia, and exposure to ototoxic medications (ref 6,7). In addition, they are exposed to high noise levels for extended periods of time (ref 8). Taken together, these conditions can all contribute to both early and late onset hearing loss, with a compounded risk in VLBW preterm infants due to extended hospital stays (ref 3).

SNHL is a permanent hearing loss that results from damage to the inner ear or cochlea, auditory nerve, and central nervous system. CHL occurs when sound cannot reach the inner ear anatomy. The result is a hearing impairment, most commonly due to transient fluid within the middle ear. NICU infants are at higher risk for both SNHL and CHL in contrast to infants in the well-baby nursery (ref 9,10). Roth and colleagues (ref 11) demonstrated a nearly ten-fold higher rate of CHL vs SNHL (2.7% vs 0.3%, respectively) in VLBW infants tested prior to NICU discharge. In addition, 22–54% of preterm infants with bronchopulmonary dysplasia developed CHL post discharge, reinforcing the need for continued close monitoring (ref 12, 13).

In contrast to previous inclusive guidelines, targeted monitoring, based on defined risk factors, seems to be a more efficient approach. A minimum NICU length-of-stay of 2 days requiring a biannual follow up schedule, imposed significant challenges particularly for rural and off-island communities (ref 4). More data is needed to better define the impact of late onset HL monitoring in the NICU population, particularly in patients with CHL, regarding language, behavioral and cognitive development.

Our follow-up standardized testing protocol resulted in a total rate of late onset hearing loss more than double the previously noted rate. As early HL detection with early intervention can have significant impact on communication development, it is important to assess the rate of referral and subsequent testing to ensure patients receive appropriate and timely care (ref 14, 15, 16). Another significant finding of this study was the high rate of late onset CHL found in VLBW NICU infants by one-year corrected age. The identification of infants with CHL increased nearly seven-fold, even at a modest patient return evaluation rate of 71%. Moreover, we also found that the incidence of CHL was nearly seven-fold the rate of late onset SNHL, although due to the one-year follow-up window, we were unable to differentiate infants with pure CHL from those with an underlying SNHL and a temporary conductive component.

Although CHL is often reversible with treatment, the deleterious effect on speech and language development should not be under-estimated, particularly if hearing is impaired during the critical period of language development (ref 17,18). High risk VLBW infants, prone to serious acute and chronic morbidities related to preterm birth, are particularly vulnerable to additional disruptions in language development. In a meta-analysis evaluating the effect of prematurity on the development of language ability, very preterm (< 32 weeks) or VLBW infants performed below control peers in both expressive and receptive language, which persisted to school age (ref 16). In a more recent report, compared to term infant controls, very preterm infants exhibited generalized language deficits that persisted through 13 years of life, with marked difficulties in expressive language and lacking signs of catch-up (ref 19).

In very preterm infants, these deficits in language comprehension and expression may be precursors to difficulties with higher functioning, including in areas of cognition, attention, academic achievement, and behavior (ref 20, 21). It is therefore essential that ongoing monitoring for late onset HL of all types with timely intervention is key to optimizing language development and overall developmental outcomes in preterm infants.

Finally, in this study, congenital CMV infection was the only risk factor found to be significantly associated with late onset hearing loss. Other studies have identified additional risk factors that can impact late onset HL, however due to the limited size of this study population, no risk factors were identified to distinguish late from early onset loss.

In conclusion, standardized monitoring for late onset HL in VLBW infants increased the identification of both SNHL and CHL. A targeted approach allowed for the highest risk infants to be monitored earlier, particularly in the face of limited pediatric audiology resources. It is likely that other therapies and complications of prematurity associated with ototoxic risk, will be identified as new treatments unfold and are introduced in the neonatal intensive care. This further reinforces the need for close follow-up of these high-risk infants. Future directions also include further definition of the language and developmental impact of late onset HL monitoring in the NICU population.

## Figures and Tables

**Table 1:** Guideline comparison: Perinatal risk factor-based monitoring for late onset hearing loss

Perinatal Risk factor	2007 JCIH recommended diagnostic follow-up	2010 KMCWC recommended diagnostic follow-up	2019 JCIH recommended diagnostic follow-up
In utero infection with CMV cytomegalovirus	By 24–30 months; monitor speech and language	Diagnostic ABR by 3 months of age	By 3 months
Culture positive infections associated with sensorineural hearing loss, including confirmed bacterial or viral (herpes virus or varicella) meningitis or encephalitis		OAE and tympanometry testing by 3 months adjusted age	
Certain birth conditions -Craniofacial malformation including microtia/atresia, ear dysplasia, oral facial clefting, white forelock, microphthalmia -Congenital microcephaly, congenital or acquired hydrocephalus -Temporal bone abnormalities			By 9 months
Extracorporeal membrane oxygenation (ECMO)		Behavioral hearing testing at 7 months corrected age	By 3 months after occurrence
In utero infection (Non CMV), such as herpes, rubella, syphilis, toxoplasmosis			By 9 months
Hyperbilirubinemia with exchange transfusion			
Asphyxia or hypoxic ischemic encephalopathy			
Aminoglycoside administration for more than 5 days			
Mechanical ventilation > 10 days			
Persistent pulmonary hypertension requiring high frequency vent or inhaled Nitric Oxide			
< or = 30 weeks gestation at birth or </= 1500 gm birth weight			
Family history of early, progressive, or delayed onset permanent childhood hearing loss			
Syndromes with progressive HL/neurodegenerative disorders			
Neonatal intensive care of more than 5 days		By 24–30 months of age; monitor speech and language	

**Table 2:** Presence of risk factors for late onset hearing loss in the VLBW infant population

Risk Factor	Late onset loss % (n = 83)	Controls % (n = 179)	p value^1^
Family history of hearing loss	2.4	0.6	ns
Craniofacial anomaly	2.4	1.7	ns
Congenital infection	4.8	0	< 0.01
Renal insufficiency^2^	6.0	11.7	ns
Severe Retinopathy of Prematurity^3^	9.6	6.2	ns
Sepsis^4^	30.1	30.7	ns
Persistent pulmonary hypertension	0	3.9	ns
Patent Ductus Arteriosus	49.4	38.0	ns
Chronic Lung Disease^5^	22.9	19.6	ns
Loop diuretic exposure	22.9	20.1	ns
Aminoglycoside exposure	60.2	58.1	ns
Gentamicin Trough > 1 mcg/mL	4.8	1.7	ns

1 p value < 0.05 considered significant

2 Renal insufficiency defined as creatinine level > 1 mg/dL

3 Severe Retinopathy of Prematurity defined as stage 3 or greater in at least one eye

4 Sepsis defined as culture positive

5 Chronic lung disease defined as oxygen requirement at 36 weeks corrected gestational age
